# Sexual activities of pregnant women attending antenatal clinic of a tertiary hospital in North-West Nigeria

**DOI:** 10.11604/pamj.2020.37.140.25471

**Published:** 2020-10-08

**Authors:** Oche Mansur Oche, Zainab Abdullahi, Karima Tunau, Jessica Timane Ango, Musa Yahaya, Ismail Abdullateef Raji

**Affiliations:** 1 Department of Community Medicine, Usmanu Danfodiyo University Teaching Hospital, Sokoto, Nigeria,; 2 Department of Community Health, Usmanu Danfodiyo University, Sokoto, Nigeria,; 3 Department of Obstetrics and Gynaecology, Usmanu Danfodiyo University Teaching Hospital, Sokoto, Nigeria,; 4 Nigeria Field Epidemiology and Laboratory Training Program, Asokoro, Abuja, Nigeria

**Keywords:** Sexual activities, pregnancy, coitus, Nigeria

## Abstract

**Introduction:**

myths about sex during pregnancy harming fetus and leading to preterm labor or miscarriage are very strong factors releasing fear and leading to avoidance of sexual contact during gestation. We therefore evaluated the attitude, sexual experiences and changes in sexual function during pregnancy.

**Methods:**

a cross-sectional study was conducted among 170 pregnant women who were selected using systematic sampling. Data were collected using an interviewer-administered questionnaire. Data was analysed using IBM SPSS® version 22.0. Descriptive statistics, Chi-square test and Cochran´s Q-test were estimated.

**Results:**

the mean age of respondents was 27.2 ± 6.2 years. Most of the respondents, 107 (62.7%) had formal education. One-fifth of the respondents, 34 (20.2%) have been married for over 10 years. More than half of them were multiparous, 112 (68.3%) and in the third trimester of pregnancy, 99 (59.6%). Majority of the respondents, 153 (87.9%) thought coitus was safe in pregnancy. More than half 89 (58.2%) had coitus at least thrice a week before pregnancy and 98.8% have engaged in sexual activities during pregnancy. Most of the respondents, 105 (61.1%) enjoyed coitus during pregnancy. The desire for coitus significantly reduced in the third trimester, p=0.001.

**Conclusion:**

sexual intercourse during pregnancy was universal and respondents engaged in sexual activities during different stages of pregnancy. Although sexual frequency declined in pregnancy compared to pre-pregnancy period, most of the respondents desired and enjoyed it. We recommend that couples are well educated to understand the normal fluctuations in sexual interest and practices during pregnancy.

## Introduction

Pregnancy is one of the special moments associated with significant hormonal changes as well as physical changes in a woman's life [1]. During this time of changes, sexuality and sexual activity are influenced by many factors, including religious beliefs and cultural influences [2]. As the pregnancy progresses, sexuality and sexual behavior are equally influenced by social, biological and psychological factors-critical components of health and well-being in a woman's life [3]. Since ancient times, there are diverse views on the sexual relationship during pregnancy; some have cautioned it or even prohibited and various studies conducted on the issue have presented diverse opinions. However, in recent times, the world has witnessed scientific interest in sexuality and sexual behavior, especially during the gestational period, vis-a-vis the associated prevalence of sexual dysfunction [4,5]. Studies have shown that about 40 to 50% of women in the general population may have some forms of sexual dysfunction sometimes in their lifetime [6]. During the pregnancy period, physiological changes do occur, leading to psychological and emotional changes that significantly differ from non-pregnant status. Sexual activity changes with apparent regression in the frequency of sexual intercourse, sexual desire, sexual satisfaction and achieving orgasm [3,7].

There are myths and misconceptions about sexual intercourse during pregnancy: perceived harm to the fetus, preterm labor, and miscarriage-are all critical factors associated with fear and anxiety and subsequent avoidance of sexual activity during pregnancy [8]. For many women, as pregnancy advances to the last stage, sexual activities such as desire, frequency and satisfaction are significantly reduced compared with other periods before pregnancy [8-13]. Possible factors responsible for such a decline in sexual activity include physical discomfort and ineptness, lack of interest, fear of injury to the fetus, painful coitus and perceived unattractiveness [14-16]. Physical and psychological factors have been found responsible for these changes [17,18]. Hormonal changes are caused by the increased secretion of estrogen, progesterone and prolactin hormones and are responsible for breast tenderness and nausea. In addition to anxiety and fatigue, exhaustion contributes to the general weakness and lack of interest in sexual activity-desire and arousal with the consequent decrease in sexual practice [19]. Sexual desire and arousal influence sexual satisfaction [19] as well as the frequency of intercourse; therefore, it is understandable why sexual practices decreased during pregnancy. In some societies, sexual intercourse during pregnancy or menstruation is prohibited and mostly presented as dangerous, which can provoke impotence, sterility, or produce monsters [20]. On the other hand, some women in Nigeria believe that sexual relations during pregnancy are beneficial, as they are supposed to widen the vagina canal, facilitating labor and delivery. The same fact can be observed in Japan, where some pregnant women believe that exhausting exercises like sexual intercourse can lighten delivery [21].

In the same vein, other studies have equally reported women's interest in sexual activity during pregnancy with the following perceived advantages: widening of the birth canal and facilitation of labor and fetal well-being [22,23]. However, other women reported sexual discomfort when pregnant [15,22]. In other studies, sexual intercourse during pregnancy was found to be associated with potential preterm labor [24,25]. Orgasm during sexual intercourse is associated with the release of oxytocin, which can initiate contractions of the uterus [26]. Also, prostaglandin's presence in the seminal fluid has been shown to have oxytocin effects [26]. During pregnancy, sexual intercourse can predispose the pregnancy to sexually transmitted diseases and consequent preterm labor [27,28]. Regardless of all the potential risks, some pregnant women give in to sexual intercourse to keep their husbands around and prevent any form of marital unfaithfulness.

This study set out to determine the attitude, sexual experiences and changes in sexual function of women during pregnancy in Sokoto metropolis. In some societies, sexual intercourse during pregnancy or menstruation is prohibited and mostly presented as dangerous, which can provoke impotence, sterility, or produce monsters [20]. On the other hand, some women in Nigeria believe that sexual relations during pregnancy are beneficial, as they are supposed to widen the vagina canal, facilitating labor and delivery. The same fact can be observed in Japan, where some pregnant women believe that exhausting exercises like sexual intercourse can lighten delivery [21]. In the same vein, other studies have equally reported women's interest in sexual activity during pregnancy with the following perceived advantages: widening of the birth canal and facilitation of labor and fetal well-being [22,23]. However, other women reported sexual discomfort when pregnant [15,22]. In other studies, sexual intercourse during pregnancy was found to be associated with potential preterm labor [24,25]. Orgasm during sexual intercourse is associated with the release of oxytocin, which can initiate contractions of the uterus [26]. Also, prostaglandin's presence in the seminal fluid has been shown to have oxytocin effects [26]. During pregnancy, sexual intercourse can predispose the pregnancy to sexually transmitted diseases and consequent preterm labor [27,28]. Regardless of all the potential risks, some pregnant women give in to sexual intercourse to keep their husbands around and prevent any form of marital unfaithfulness. This study set out to determine the attitude, sexual experiences and changes in sexual function of women during pregnancy in Sokoto metropolis.

## Methods

A descriptive cross-sectional study was conducted among pregnant women attending the Antenatal Clinic (ANC) of Usmanu Danfodiyo University Teaching Hospital (UDUTH) Sokoto, Nigeria, between March and June, 2019. The hospital has a bed capacity of 800 and serves Sokoto State and the neighboring States of Kebbi and Zamfara, as well as Niger republic. The hospital carries out curative, preventive and rehabilitative services in addition to being the regional center for neurosurgery. The ANC is one of several clinics under the Obstetrics and Gynecology Department and runs from Monday to Friday seeing new, old and referred cases (the other clinics include Feto-maternal, Oncology and Urogynecology). All pregnant women attending the antenatal clinic of UDUTH within the period of the study and gave their informed consent to participate were considered eligible and enrolled in the study. The women who were very ill or going into labor were excluded from the study (exclusion criteria). Using the Fisher´s formula for estimating sample size for descriptive studies [29], at a precision level of 5% and an anticipated participant response rate of 95%, a sample size of 176 was obtained. Using the clinic´s daily attendance register, the eligible participants were selected by systematic sampling technique; one of four women presenting consecutively at the antenatal clinic of the hospital and met the eligibility criteria was enrolled into the study over a period of two months until the required sample size was obtained.

**Data collection:** a standardized, semi-structured, self and interviewer-administered questionnaire was used to obtain information on the study participants' socio-demographic characteristics, obstetric history, attitude towards sexuality in pregnancy and sexual experiences of women before and during pregnancy. The questionnaire was pretested among 20 women attending the antenatal clinic of the Sokoto State specialist hospital, Sokoto, which is a tertiary hospital within the metropolis. Four resident doctors from the Departments of Community Medicine and Obstetrics and Gynaecology of UDUTH were trained on the objectives of the study, interpersonal communication and conduct of survey research and subsequently assisted in questionnaire administration. Data were analysed using IBM SPSS computer software version 22 after ensuring complete entry. Frequencies, proportions, mean and standard deviation were used to describe the socio-demographic profile, obstetric history, sexual experiences and attitude towards sexuality in pregnancy. Chi-square test was used to determine the association between sexual activities in pregnancy and other factors. Cochran´s Q-test was used to determine if there is a significant change in sexual activities across the three trimesters of pregnancy. All statistical analyses were performed at 5% level of significance. Ethical approval for this study was obtained from the Ethics and Research Committee of the Usmanu Danfodiyo University Teaching Hospital, Sokoto and informed written consent was obtained from all consenting participants before the commencement of data collection. Permission was also gotten from the Head, Obstetrics and Gynaecology Department of UDUTH.

## Results

The mean age of respondents was 27.2 ± 6.2 years with majority in (156 (90.7%)) 26-35-year age group. More than two-third were muslims (142 (81.1%)) and almost all were married (169 (96.6%)). Only 16 (9.4%) of the respondent have primary education (Table 1). One-fifth of the respondents (34 (20.2%)) have been married for over 10 years. More than half of them were multiparous (112 (68.3%)) and in the third trimester of pregnancy (99 (59.6%)). Almost two-third had no prior history of abortion (124 (72.5%)) and more than two-third had no complication in pregnancy (136 (78.2%)) (Table 2). Majority of the respondents 153 (87.9%) thought coitus was safe in pregnancy, mainly because it does not cause any harm to them and baby 121 (78.1%), keeps the man around the home 88 (56.8%) and it enhances partner´s connection 83 (53.5%) (Table 3). Most of the respondents 89 (58.2%) had coitus at least thrice a week before pregnancy. The prevalence of ever had coitus during pregnancy was 98.8%. Majority of the respondents 113 (64.9%) desired coitus during pregnancy, however, barely half of them 71 (43.6%) had sexual intercourse once a week during pregnancy. The main reasons were to satisfy husband´s desire 128 (76.2%), an important marital obligation 117 (69.6%). Majority of the respondents 147 (85.5%) will recommend coitus during pregnancy (Table 4). Majority (146 (86.4)) of the respondents that have ever had coitus during pregnancy will recommend coitus during pregnancy (Fisher´s exact test, p=0.021) (Table 5).

**Table 1:** socio-demographic profile of women attending antenatal clinic in a tertiary hospital in North-Western Nigeria, 2019

Variables	Frequency (%)
**Age in years**	
≤25	76 (44.2)
26 – 35	80 (46.5)
≥36	16 (9.3)
Total	172 (100)
**Religion**	
Islam	142 (81.1)
Christianity	30 (17.1)
Others	3 (1.7)
Total	175 (100)
**Marital status**	
Single	6 (3.4)
Married	169 (96.6)
Total	175 (100)
**Tribe**	
Hausa	106 (60.9)
Fulani	16 (9.2)
Yoruba	11 (6.3)
Igbo	18 (10.3)
Others	23 (13.2)
Total	174 (100)
**Educational Status**	
None	7 (4.1)
Quranic only	40 (23.5)
Primary	16 (9.4)
Secondary	40 (23.5)
Tertiary	67 (39.4)
Total	170 (100)
**Occupation**	
None	6 (4.0)
Student	12 (8.0)
House wife	62 (41.3)
Civil servant	32 (21.3)
Business	38 (25.3)
Total	150 (100)

**Table 2:** obstetric history of women attending antenatal clinic in a tertiary hospital in North-Western Nigeria, 2019

Variables	Frequency (%)
**Duration of marriage in years**	
<5	78 (46.4)
5 - 10	56 (33.3)
>10	34 (20.2)
Total	168 (100)
**Parity**	
Primipara	52 (31.7)
Multipara	112 (68.3)
Total	164 (100)
**History of abortion**	
No	124 (72.5)
Yes	47 (27.5)
Total	171 (100)
**Stage of pregnancy**	
First trimester	23 (13.9)
Second trimester	44 (26.5)
Third trimester	99 (59.6)
Total	166 (100)
**Complication in pregnancy**	
No	136 (78.2)
Yes	38 (21.8)
Total	174 (100)
**Period of complication**	
First trimester	3 (8.3)
Second trimester	31 (86.1)
Third trimester	2 (5.6)
Total	38 (100)
**Complications**	
Bleeding	4 (16.0)
Anaemia	4 (16.0)
Malaria	3 (12.0)
Pulmonary embolism	1 (4.0)
Excessive vomiting	1 (4.0)
Hypertensive disorders	2 (8.0)
Severe LAP	2 (8.0)
Lower abdominal pain	2 (8.0)
Threatened abortion	2 (8.0)
Abdominal pain	1 (4.0)
Too much fluid	1 (4.0)
Vomiting	1 (4.0)
UTI	1 (4.0)
Total	25 (100)

**Table 3:** attitude towards sexuality in pregnancy among women attending antenatal clinic in a tertiary hospital in North-Western Nigeria, 2019

Variables	Frequency (%) n=174
**Sexual activities in pregnancy is safe**	
No	21 (12.1)
Yes	153 (87.9)
If yes, reasons^*^	
Does not cause any harm to me and baby	121 (78.1)
Enhances partners connection	83 (53.5)
Strengthens genital muscles	46 (11.3)
Will keep the man around the home	88 (56.8)
Widens the vagina and thus makes labour easy	70 (45.2)
If no, worries^*^	
Abnormal	2 (18.2)
Forbidden	1 (9.1)
Pain	8 (72.7)
Discomfort	8 (72.7)
Bleeding	6 (54.5)
Miscarriage	8 (72.7)
Preterm labour	2 (18.2)
Injury to the baby	3 (27.3)
Genital infection	1 (9.1)
Infection to the baby	1 (9.1)
Others	1 (9.1)
**Stage of pregnancy you worry most**	
First trimester	60 (40.3)
Second trimester	10 (6.7)
Third trimester	79 (52.0)
Total	149 (100)
**Physical appearance of a woman during pregnancy hinders sexual activities**	
Yes	66 (39.3)
No	102 (60.7)
Total	168 (100)
**Disorders of pregnancy should hinder sexual activities**	
Yes	88 (53.7)
No	76 (46.3)
Total	164 (100)

* Multiple response

**Table 4:** sexual experiences before and during pregnancy among women attending antenatal clinic in a tertiary hospital in North-Western Nigeria, 2019

Variables	Frequency (%)
**Incidence of sexual intercourse before pregnancy**	
Once a week	21 (13.7)
Twice a week	43 (28.1)
≥thrice a week	89 (58.2)
Total	153 (100)
**Ever had sexual intercourse during any trimester of pregnancy**	
Yes	171 (98.8)
No	2 (1.2)
Total	173 (100)
**Desire for sexual intercourse during pregnancy**	
Yes	113 (64.9)
No	61 (35.1)
Total	174 (100)
**Frequency of coitus during pregnancy**	
Once a week	71 (43.6)
Twice a week	46 (28.2)
thrice a week	46 (28.2)
Total	163 (100)
Reasons for having coitus during pregnancy*	
It brings marital harmony	113 (67.3)
To satisfy my urge	45 (26.8)
Satisfy husband's desire	128 (76.2)
Show of love	48 (28.6)
Marital obligations	117 (69.6)
Keeps husbands around and prevents infidelity	99 (58.9)
Widens the birth canal and facilitates easy delivery	62 (36.9)
I attain orgasm much easily	14 (8.3)
**Change in sexual functioning during pregnancy**	
Yes	98 (57.3)
No	73 (42.7)
Total	171(100)
**Enjoys coitus during pregnancy**	
Yes	105 (61.0)
No	67 (39.0)
Total	172 (100)
Reasons for not having coitus*	
Pain	41 (41.8)
Discomfort	65 (66.3)
Bruises and cuts	4 (4.1)
Bleeding	5 (5.1)
Miscarriage	2 (2.0)
Preterm labour	3 (3.1)
Genital infection	1 (1.0)
Infection to the baby	0 (0.0)
Others**	9 (9.2)
**Advise over coitus during pregnancy**	
Yes	147 (85.5)
No	24 (14.5)
Total	171 (100)
**Advise reduced frequency of coitus during pregnancy**	
Yes	120 (75.9)
No	31 (19.6)
Indifferent	7 (4.4)
Total	158 (100)

* Multiple response analysis; **Nausea and vomiting

**Table 5:** factors affecting sexuality during pregnancy among women attending antenatal clinic in a tertiary hospital in North-Western Nigeria, 2019

Variables	Have you ever had coitus in pregnancy		Test statistics, p-values
	No	Yes	
	Frequency (%)	Frequency (%)	
**Age of respondents in years**			
<25	1 ((50.0)	75 (44.6)	LR = 398
26-35	1 (50.0)	77 (45.8)	p = 0.819
≥36	0 (0.0)	16 (9.5)	
**Educational status**			
Informal (primary and below)	2 (100.0)	44 (26.5)	Fisher's Exact
Formal (secondary and above)	0 (0.0)	122 (73.5)	p = 0.074
**Occupational status**			
None	0 (0.0)	6 (4.1)	LR = 3.56
Student	0 (0.0)	12 (8.2)	p = 0.465
House wife	2 (100.0)	59 (40.4)	
Civil servant	0 (0.0)	31 (21.2)	
Business	0 (0.0)	38 (26.0)	
**Duration of marriage in years**			
<5	1 (50.0)	76 (46.3)	LR=0.944
5-10	1 (50.0)	55 (33.5)	p=0.624
>10	0 (0.0)	33 (20.1)	
**Stage of pregnancy**			
First trimester	2 (100.0)	21 (13.0)	LR=8.0
Second trimester	0 (0.0)	44 (27.2)	P=0.018
Third trimester	0 (0.0)	97 (59.9)	
**Recommend coitus during pregnancy**			
No	2 (100.0)	23 (13.6)	Fisher's Exact
Yes	0 (0.0)	146 (86.4)	p=0.021

The desire for coitus was significantly different before pregnancy and at any stage of pregnancy (Q=83.63, p<0.001). The desire for coitus was significantly reduced in the third trimester than second (p=0.001), first trimester (p<0.001) and before pregnancy (p<0.001) according to Dunn´s test. Acceptance to have coitus initiated by partner differed before pregnancy and at any stage of pregnancy (Q=11.61, p=0.009). This was significantly higher before pregnancy compared with any stage of pregnancy according to Dunn´s test (p=0.005). The responses for male dominant position was significantly different before pregnancy and any stage of pregnancy (Q=25.02, p<0.001). This was lower in the third trimester compared with first trimester (p=0.004) and before pregnancy (p<0.001) and second trimester compared with before pregnancy according to Dunn´s test (p=0.048). Achievement of coital satisfaction significantly reduced over time during pregnancy (Q=87.18, p<0.001). It was significantly higher before pregnancy compare with first trimester (p=0.004), second trimester (p<0.001), third trimester (p<0.001) and between first trimester compared with second trimester (p=0.012) and third trimester (p<0.001) (Table 6).

**Table 6:** changes in sexual functioning before and during pregnancy among women attending antenatal clinic in a tertiary hospital in North-Western Nigeria, 2019

Variables	Before pregnancy	Trimester			Test statistics^*^
		1^st^	2^nd^	3^rd^	
	Frequency	Frequency	Frequency	Frequency	
**Desire for coitus**					
Yes	121	88	86	60	Q=83.63
No	9	42	44	70	p<0.001^**^
**Partner initiation acceptable**					
Yes	134	126	129	121	Q=11.61
No	8	16	13	21	p=0.009^**^
**Male dominated sexual positions**					
Yes	116	110	105	96	Q=25.02
No	9	15	20	29	p<0.001^**^
**Female dominated sexual positions**					
Yes	75	60	61	52	Q=11.30^**^
No	50	65	56	20	p=0.010
**Dyspareunia**					
Yes	14	26	32	45	Q=37.64
No	131	119	113	100	p<0.001^**^
**Self-sexual satisfaction**					
Yes	131	111	93	80	Q=87.18
No	12	32	50	63	p<0.001^**^
**Partner's sexual satisfaction**					
Yes	141	139	138	131	Q=20.64
No	4	6	7	14	p<0.001

* Cochran's Q-test

## Discussion

During the different phases of women´s lives, transformations occur in their bodies, making them feel less sensual and less sexually attractive, which does not correspond to the culturally disseminated aesthetic pattern [30]. In this study, more than half of the respondents opined that they desired sexual intercourse during pregnancy. This is in contrast to the study among Iranian women which found a decline in their sexual desires [2]; findings from other studies also indicated a decline in sexual desire amongst their respondents [2,13-15,31,32]. The decline in sexual desire among women generally has been attributed to the physiological, psychological and emotional changes that take place during pregnancy [8,13,33]. In this study, sexual intercourse in pregnancy was universal as almost all of the women had sexual intercourse at any stage of their pregnancies. In the study among Hong Kong Chinese women, more than one third stopped vaginal sexual intercourse and this was much higher than 11 and 14% observed in the United States of America and Canada respectively [33,34]. In a meta-analysis of 59 studies, predominantly conducted in USA and Europe, only 10% of the women abstained from coitus once pregnancy was confirmed [35]. The mean frequency of intercourse during pregnancy in our study (1.99 times/week) was less than before pregnancy (2.97 times/week). This is in consonance with findings from similar studies where coital frequencies decreased during pregnancy [15,22,23,36].

Findings from this study showed that the most challenging period for the respondents was during third trimester as reported by more than half of the women (52%). The level of emotional stress and anxiety related to the possible complications increases as the time of delivery approaches [36-38]. The woman feels less attractive as a sexual partner because of increasing fatigue, dyspnea, edema, contractions and general physical exhaustion [7,8,39,40]. In a study by Onah *et al*. the frequency of coitus was reported to be decreased from 3.2 per week before pregnancy to 1.8 per week during pregnancy [32]. However, these findings are in sharp contrast to the findings from other studies which observed an increase in the frequency of intercourse during pregnancy associated with a rise in uterine activities, acceleration of onset of labor and prevention of prolonged pregnancy thus reducing the need for induction [41,42]. Some other studies demonstrated a relative increase in sexual functions during second trimester when compared to the first and third trimesters [43,44]. The male dominant position was significantly more before pregnancy than any other stages of pregnancy. One of the commonest reasons why women in our study indulged in sexual intercourse during pregnancy was to satisfy their husbands. This finding is in consonance with the findings from similar studies [15,22,23]. A meta-content analysis of 59 studies on sexuality during pregnancy and after childbirth noted that “female coital activity is often motivated by a concern about the partner (fulfilling marital obligations, concern about the sexual satisfaction of the partner); this plays a role in every phase of parenthood-for example, the first intercourse after childbirth” [35]. It may be correct therefore to infer, as well, that if sexual frequency and satisfaction can be improved during pregnancy, partners may be less likely to engage in extramarital affairs, thus reducing all its inherent hazards [45]. More than a quarter (44.3%) of the respondents opined that they consented to intercourse in order to widen the vagina and facilitate labor. Similar reasons were found in other studies to justify keeping sex during pregnancy [15,22,23].

In our study, pregnant women who kept sex gave the following reasons: show of love, brings about marital harmony, satisfy my sexual urge and attainment of orgasm. Of note in this study is the concern pregnant women had concerning sexual intercourse in pregnancy that could lead to obstetrics complications. A greater majority of women in Northern Nigeria live in family settings where the influences of mothers and grandmothers are overwhelming with women holding high perceptions about certain cultural beliefs and practices. Although majority of the beliefs were a product of culturally held views, in some cases they could also have been influenced by remnants of fears/concerns from poor obstetric experiences in the past [23]. Some of the fears expressed by our respondents included, causing abortion preterm labor and injuries to the baby in utero which are in tandem with findings from other studies [9,33,43,44]. Bartellas *et al*. reported that 49% of women worried that sexual activity could possibly harm their pregnancies with concerns usually regarding premature delivery or preterm premature rupture of membranes high in their minds [33]. Amongst the Ewe in Togo, the husband who transgressed the prohibition of sexual intercourse during pregnancy might cause a miscarriage or a stillbirth child [45]. This again reflects the conflict and guilt women feel between deciding to keep the baby from harm and pressures to fulfill marital obligations [46]. In contrast to these findings, among the Azanda tribe in the Democratic Republic of Congo, the sperm is considered as an important factor in the growth of the fetus [47]; also among the Dogon in Mali, it is necessary to have sex with the expectant mother for the proper growth of the child [48].

Most of the women who expressed worries about sexual intercourse in pregnancy attributed it to dyspareunia. This may have a negative effect on marital bonds and may be an obstacle for the adaptation of women to this transient phase. Several studies have documented varying degrees of dyspareunia during pregnancy, similar to the findings in our study [9,15,22,49]. Other studies reported women abandoning sexual intercourse during pregnancy as a result of severe pain as confirmed by the reports of Hanafy *et al*. and Chang *et al*. [7,39]. However, these beliefs and perceptions, wittingly or unwittingly are contrary to scientific knowledge. In fact, no study so far has shown danger of sexual intercourse on a normal pregnancy [50]. Majority of the respondents in our study opined that they would recommend sexual intercourse in pregnancy to other women, although with reduced frequency. This is in keeping with findings from other studies [25]. Findings from our study also showed that majority of the women believed that coitus was safe and actually enjoyed having it during pregnancy. The achievement of coital satisfaction significantly reduced over time during pregnancy. This is in contrast with findings from a similar study in Poland where only 3.6% of their respondents expressed satisfaction with sexual activities during pregnancy [51]. Despite the misbeliefs and misperceptions about sexual intercourse during pregnancy, majority of the respondents that have ever had coitus during pregnancy will recommend coitus during pregnancy, which may not be unconnected to their desire to maintain marital harmony and limit or prevent spousal infidelity. This is in tandem with findings from a similar study in Nigeria [22]. Many demographic factors such as age, educational level, parity and duration of marriage were suggested to affect sexuality during pregnancy. Although some studies demonstrated significant correlation between age and sexuality in pregnancy, others failed to show this relationship [37,43,50,52].

A study with 220 muslim women reported that younger age, multi-parity and lesser duration of marriage were positively correlated with female sexuality [53]. The relationship between educational level and sexual functions was also found to be controversial [12,17,53,54]. Gottlieb also demonstrated that people who have attained university education are less likely to experience sexual problems than their less well-educated counterparts [55]. Bivariate analysis showed that only stage of pregnancy and recommending coitus during pregnancy was significantly associated with coitus during pregnancy. In the midst of misinformation concerning sexuality during pregnancy, a woman often gets major part of her information from other fellow women and all sorts of misconceptions may be delivered. Educated women were thought to tend to search other information sources about sexuality and by this way, prevent themselves from misbeliefs which were found to be the reasons of sexual dysfunction during pregnancy. Besides, some authors reported that low educational level was also related to the increased number of pregnancies in the adolescent period [56,57]. Those adolescents begin their sexual life early, carry a risk of unplanned pregnancy as a result of lack of opportunities of access to information about sexuality, in addition to search for pleasure, absence of guidance and advice of families, natural curiosity and need for self-affirmation. Our present study however did not exhibit any significant association between sexuality in pregnancy and low educational level.

## Conclusion

This study observed that sexual intercourse during pregnancy was universal as about 99% of the respondents engaged in sexual activities during different stages of pregnancy. Although sexual frequency declined in pregnancy compared to pre-pregnancy period, most of the respondents desired and actually enjoyed doing it. The decline in sexual activity was associated with fear of harm to the foetus and premature labor. However, the women who kept sex did so as a show of love for partners, ensuring marital harmony and also to satisfy their sexual urge. Given that sexual matters are private in nature, it is imperative that attending physicians or midwives discuss it during individual examination time, thus encouraging the client to communicate more freely in an open manner; this means emphasis should be on doctor-initiated counseling. In most African nay Nigerian societies where behaviors are molded by cultures that are in most cases shrouded in myths and misconceptions, it is important that physicians and midwives dispel such myths and reassure the women and their partners that sexual intercourse during pregnancy in the majority of couples will not lead to any adverse effects on the pregnancy except where there are potential threats in which the physician will advise accordingly. It is also important that couples are well educated to understand the normal fluctuations in sexual interest and practices and that a progressive decline in sexual desire is more common in women than in men as pregnancy progresses.

### What is known about this topic

- Sexual activities such as desire, frequency and satisfaction are significantly reduced compared with other periods before pregnancy;
- Factors responsible for such a decline in sexual activity include physical discomfort and ineptness, lack of interest, fear of injury to the fetus, painful coitus and perceived unattractiveness.

### What this study adds

- Sexual experience of women in a conservative environment ascertained;
- Women maintain their sexual relationships as show of love to partners and to maintain marital harmony.
